# Supplementary material for: HIVprotI: an integrated web based platform for prediction and design of HIV proteins inhibitors
Source: J Cheminform. 2018 Mar 9;10:12. doi: 10.1186/s13321-018-0266-y (PMC5845081; doi:10.1186/s13321-018-0266-y)
Supplement: Supplementary file 2 — Additional file 2. Source code of HIVProtI web server. [file 13321_2018_266_MOESM2_ESM.zip › HIVprotI_Source-code/hivprotI/weka-3-6-6/doc/weka/classifiers/meta/RegressionByDiscretization.html]

RegressionByDiscretization


---


|  |  |  |
| --- | --- | --- |
| **Overview** \| **Package** \| **Class** \| **Tree** \| **Deprecated** \| **Index** \| **Help** \| **Weka's home** | |  |
| **PREV CLASS**   **NEXT CLASS** | **FRAMES**    **NO FRAMES**     **All Classes** |  |
| SUMMARY: NESTED \| FIELD \| CONSTR \| METHOD | DETAIL: FIELD \| CONSTR \| METHOD |  |


---


## weka.classifiers.meta Class RegressionByDiscretization

```
java.lang.Object
  weka.classifiers.Classifier
      weka.classifiers.SingleClassifierEnhancer
          weka.classifiers.meta.RegressionByDiscretization
```

**All Implemented Interfaces:**: java.io.Serializable, java.lang.Cloneable, CapabilitiesHandler, OptionHandler, RevisionHandler

---

``` public class RegressionByDiscretization extends SingleClassifierEnhancer ```

A regression scheme that employs any classifier on a copy of the data that has the class attribute (equal-width) discretized. The predicted value is the expected value of the mean class value for each discretized interval (based on the predicted probabilities for each interval).


Valid options are:

```
 -B <int>
  Number of bins for equal-width discretization
  (default 10).
```

```
 -E
  Whether to delete empty bins after discretization
  (default false).
```

```
 -F
  Use equal-frequency instead of equal-width discretization.
```

```
 -D
  If set, classifier is run in debug mode and
  may output additional info to the console
```

```
 -W
  Full name of base classifier.
  (default: weka.classifiers.trees.J48)
```

```
 Options specific to classifier weka.classifiers.trees.J48:
```

```
 -U
  Use unpruned tree.
```

```
 -C <pruning confidence>
  Set confidence threshold for pruning.
  (default 0.25)
```

```
 -M <minimum number of instances>
  Set minimum number of instances per leaf.
  (default 2)
```

```
 -R
  Use reduced error pruning.
```

```
 -N <number of folds>
  Set number of folds for reduced error
  pruning. One fold is used as pruning set.
  (default 3)
```

```
 -B
  Use binary splits only.
```

```
 -S
  Don't perform subtree raising.
```

```
 -L
  Do not clean up after the tree has been built.
```

```
 -A
  Laplace smoothing for predicted probabilities.
```

```
 -Q <seed>
  Seed for random data shuffling (default 1).
```

**Version:**
:   $Revision: 4746 $

**Author:**
:   Len Trigg (trigg@cs.waikato.ac.nz), Eibe Frank (eibe@cs.waikato.ac.nz)

**See Also:**: Serialized Form

---

**Constructor Summary**

`RegressionByDiscretization()`
  
          Default constructor.


| **Method Summary** | |
| --- | --- |
| `void` | `buildClassifier(Instances instances)`             Generates the classifier. |
| `double` | `classifyInstance(Instance instance)`             Returns a predicted class for the test instance. |
| `java.lang.String` | `deleteEmptyBinsTipText()`             Returns the tip text for this property |
| `Capabilities` | `getCapabilities()`             Returns default capabilities of the classifier. |
| `boolean` | `getDeleteEmptyBins()`             Gets the number of bins numeric attributes will be divided into |
| `int` | `getNumBins()`             Gets the number of bins numeric attributes will be divided into |
| `java.lang.String[]` | `getOptions()`             Gets the current settings of the Classifier. |
| `java.lang.String` | `getRevision()`             Returns the revision string. |
| `boolean` | `getUseEqualFrequency()`             Get the value of UseEqualFrequency. |
| `java.lang.String` | `globalInfo()`             Returns a string describing classifier |
| `java.util.Enumeration` | `listOptions()`             Returns an enumeration describing the available options. |
| `static void` | `main(java.lang.String[] argv)`             Main method for testing this class. |
| `java.lang.String` | `numBinsTipText()`             Returns the tip text for this property |
| `void` | `setDeleteEmptyBins(boolean b)`             Sets the number of bins to divide each selected numeric attribute into |
| `void` | `setNumBins(int numBins)`             Sets the number of bins to divide each selected numeric attribute into |
| `void` | `setOptions(java.lang.String[] options)`             Parses a given list of options. |
| `void` | `setUseEqualFrequency(boolean newUseEqualFrequency)`             Set the value of UseEqualFrequency. |
| `java.lang.String` | `toString()`             Returns a description of the classifier. |
| `java.lang.String` | `useEqualFrequencyTipText()`             Returns the tip text for this property |

**Methods inherited from class weka.classifiers.SingleClassifierEnhancer**

`classifierTipText, getClassifier, setClassifier`

**Methods inherited from class weka.classifiers.Classifier**

`debugTipText, distributionForInstance, forName, getDebug, makeCopies, makeCopy, setDebug`

**Methods inherited from class java.lang.Object**

`equals, getClass, hashCode, notify, notifyAll, wait, wait, wait`

**Constructor Detail**

### RegressionByDiscretization

```
public RegressionByDiscretization()
```

:   Default constructor.


**Method Detail**

### globalInfo

```
public java.lang.String globalInfo()
```

:   Returns a string describing classifier

    :   **Returns:**: a description suitable for displaying in the explorer/experimenter gui

---


### getCapabilities

```
public Capabilities getCapabilities()
```

:   Returns default capabilities of the classifier.

    :   **Specified by:**: `getCapabilities` in interface `CapabilitiesHandler` **Overrides:**: `getCapabilities` in class `SingleClassifierEnhancer`
    :   **Returns:**: the capabilities of this classifier **See Also:**: `Capabilities`

---


### buildClassifier

```
public void buildClassifier(Instances instances)
                     throws java.lang.Exception
```

:   Generates the classifier.

    :   **Specified by:**: `buildClassifier` in class `Classifier`
    :   **Parameters:**: `instances` - set of instances serving as training data **Throws:**: `java.lang.Exception` - if the classifier has not been generated successfully

---


### classifyInstance

```
public double classifyInstance(Instance instance)
                        throws java.lang.Exception
```

:   Returns a predicted class for the test instance.

    :   **Overrides:**: `classifyInstance` in class `Classifier`
    :   **Parameters:**: `instance` - the instance to be classified **Returns:**: predicted class value **Throws:**: `java.lang.Exception` - if the prediction couldn't be made

---


### listOptions

```
public java.util.Enumeration listOptions()
```

:   Returns an enumeration describing the available options.

    :   **Specified by:**: `listOptions` in interface `OptionHandler` **Overrides:**: `listOptions` in class `SingleClassifierEnhancer`
    :   **Returns:**: an enumeration of all the available options.

---


### setOptions

```
public void setOptions(java.lang.String[] options)
                throws java.lang.Exception
```

:   Parses a given list of options.


    Valid options are:

    ```
     -B <int>
      Number of bins for equal-width discretization
      (default 10).
    ```

    ```
     -E
      Whether to delete empty bins after discretization
      (default false).
    ```

    ```
     -F
      Use equal-frequency instead of equal-width discretization.
    ```

    ```
     -D
      If set, classifier is run in debug mode and
      may output additional info to the console
    ```

    ```
     -W
      Full name of base classifier.
      (default: weka.classifiers.trees.J48)
    ```

    ```
     Options specific to classifier weka.classifiers.trees.J48:
    ```

    ```
     -U
      Use unpruned tree.
    ```

    ```
     -C <pruning confidence>
      Set confidence threshold for pruning.
      (default 0.25)
    ```

    ```
     -M <minimum number of instances>
      Set minimum number of instances per leaf.
      (default 2)
    ```

    ```
     -R
      Use reduced error pruning.
    ```

    ```
     -N <number of folds>
      Set number of folds for reduced error
      pruning. One fold is used as pruning set.
      (default 3)
    ```

    ```
     -B
      Use binary splits only.
    ```

    ```
     -S
      Don't perform subtree raising.
    ```

    ```
     -L
      Do not clean up after the tree has been built.
    ```

    ```
     -A
      Laplace smoothing for predicted probabilities.
    ```

    ```
     -Q <seed>
      Seed for random data shuffling (default 1).
    ```

    :   **Specified by:**: `setOptions` in interface `OptionHandler` **Overrides:**: `setOptions` in class `SingleClassifierEnhancer`
    :   **Parameters:**: `options` - the list of options as an array of strings **Throws:**: `java.lang.Exception` - if an option is not supported

---


### getOptions

```
public java.lang.String[] getOptions()
```

:   Gets the current settings of the Classifier.

    :   **Specified by:**: `getOptions` in interface `OptionHandler` **Overrides:**: `getOptions` in class `SingleClassifierEnhancer`
    :   **Returns:**: an array of strings suitable for passing to setOptions

---


### numBinsTipText

```
public java.lang.String numBinsTipText()
```

:   Returns the tip text for this property

    :   **Returns:**: tip text for this property suitable for displaying in the explorer/experimenter gui

---


### getNumBins

```
public int getNumBins()
```

:   Gets the number of bins numeric attributes will be divided into

    :   **Returns:**: the number of bins.

---


### setNumBins

```
public void setNumBins(int numBins)
```

:   Sets the number of bins to divide each selected numeric attribute into

    :   **Parameters:**: `numBins` - the number of bins

---


### deleteEmptyBinsTipText

```
public java.lang.String deleteEmptyBinsTipText()
```

:   Returns the tip text for this property

    :   **Returns:**: tip text for this property suitable for displaying in the explorer/experimenter gui

---


### getDeleteEmptyBins

```
public boolean getDeleteEmptyBins()
```

:   Gets the number of bins numeric attributes will be divided into

    :   **Returns:**: the number of bins.

---


### setDeleteEmptyBins

```
public void setDeleteEmptyBins(boolean b)
```

:   Sets the number of bins to divide each selected numeric attribute into

    :   **Parameters:**: `numBins` - the number of bins

---


### useEqualFrequencyTipText

```
public java.lang.String useEqualFrequencyTipText()
```

:   Returns the tip text for this property

    :   **Returns:**: tip text for this property suitable for displaying in the explorer/experimenter gui

---


### getUseEqualFrequency

```
public boolean getUseEqualFrequency()
```

:   Get the value of UseEqualFrequency.

    :   **Returns:**: Value of UseEqualFrequency.

---


### setUseEqualFrequency

```
public void setUseEqualFrequency(boolean newUseEqualFrequency)
```

:   Set the value of UseEqualFrequency.

    :   **Parameters:**: `newUseEqualFrequency` - Value to assign to UseEqualFrequency.

---


### toString

```
public java.lang.String toString()
```

:   Returns a description of the classifier.

    :   **Overrides:**: `toString` in class `java.lang.Object`
    :   **Returns:**: a description of the classifier as a string.

---


### getRevision

```
public java.lang.String getRevision()
```

:   Returns the revision string.

    :   **Specified by:**: `getRevision` in interface `RevisionHandler` **Overrides:**: `getRevision` in class `Classifier`
    :   **Returns:**: the revision

---


### main

```
public static void main(java.lang.String[] argv)
```

:   Main method for testing this class.

    :   **Parameters:**: `argv` - the options


---


|  |  |  |
| --- | --- | --- |
| **Overview** \| **Package** \| **Class** \| **Tree** \| **Deprecated** \| **Index** \| **Help** \| **Weka's home** | |  |
| **PREV CLASS**   **NEXT CLASS** | **FRAMES**    **NO FRAMES**     **All Classes** |  |
| SUMMARY: NESTED \| FIELD \| CONSTR \| METHOD | DETAIL: FIELD \| CONSTR \| METHOD |  |


---
